# Diverging patterns of plasticity in the nucleus basalis of Meynert in early- and late-onset blindness

**DOI:** 10.1093/braincomms/fcad119

**Published:** 2023-04-11

**Authors:** Ji Won Bang, Russell W Chan, Carlos Parra, Matthew C Murphy, Joel S Schuman, Amy C Nau, Kevin C Chan

**Affiliations:** Department of Ophthalmology, NYU Grossman School of Medicine, NYU Langone Health, New York University, New York, NY 10017, USA; Department of Ophthalmology, NYU Grossman School of Medicine, NYU Langone Health, New York University, New York, NY 10017, USA; Department of Ophthalmology, NYU Grossman School of Medicine, NYU Langone Health, New York University, New York, NY 10017, USA; Department of Radiology, Mayo Clinic, Rochester, MN 55905, USA; Department of Ophthalmology, School of Medicine, University of Pittsburgh, Pittsburgh, PA 15213, USA; Department of Ophthalmology, NYU Grossman School of Medicine, NYU Langone Health, New York University, New York, NY 10017, USA; Neuroscience Institute, NYU Grossman School of Medicine, NYU Langone Health, New York University, New York, NY 10016, USA; Center for Neural Science, College of Arts and Science, New York University, New York, NY 10003, USA; Department of Biomedical Engineering, Tandon School of Engineering, New York University, New York, NY 11201, USA; Department of Ophthalmology, School of Medicine, University of Pittsburgh, Pittsburgh, PA 15213, USA; Korb and Associates, Boston, MA 02215, USA; Department of Ophthalmology, NYU Grossman School of Medicine, NYU Langone Health, New York University, New York, NY 10017, USA; Department of Ophthalmology, School of Medicine, University of Pittsburgh, Pittsburgh, PA 15213, USA; Neuroscience Institute, NYU Grossman School of Medicine, NYU Langone Health, New York University, New York, NY 10016, USA; Center for Neural Science, College of Arts and Science, New York University, New York, NY 10003, USA; Department of Biomedical Engineering, Tandon School of Engineering, New York University, New York, NY 11201, USA; Department of Radiology, NYU Grossman School of Medicine, NYU Langone Health, New York University, New York, NY 10016, USA

**Keywords:** nucleus basalis of Meynert, blindness, plasticity, choline, basal forebrain

## Abstract

Plasticity in the brain is impacted by an individual’s age at the onset of the blindness. However, what drives the varying degrees of plasticity remains largely unclear. One possible explanation attributes the mechanisms for the differing levels of plasticity to the cholinergic signals originating in the nucleus basalis of Meynert. This explanation is based on the fact that the nucleus basalis of Meynert can modulate cortical processes such as plasticity and sensory encoding through its widespread cholinergic projections. Nevertheless, there is no direct evidence indicating that the nucleus basalis of Meynert undergoes plastic changes following blindness. Therefore, using multiparametric magnetic resonance imaging, we examined if the structural and functional properties of the nucleus basalis of Meynert differ between early blind, late blind and sighted individuals. We observed that early and late blind individuals had a preserved volumetric size and cerebrovascular reactivity in the nucleus basalis of Meynert. However, we observed a reduction in the directionality of water diffusion in both early and late blind individuals compared to sighted individuals. Notably, the nucleus basalis of Meynert presented diverging patterns of functional connectivity between early and late blind individuals. This functional connectivity was enhanced at both global and local (visual, language and default-mode networks) levels in the early blind individuals, but there were little-to-no changes in the late blind individuals when compared to sighted controls. Furthermore, the age at onset of blindness predicted both global and local functional connectivity. These results suggest that upon reduced directionality of water diffusion in the nucleus basalis of Meynert, cholinergic influence may be stronger for the early blind compared to the late blind individuals. Our findings are important to unravelling why early blind individuals present stronger and more widespread cross-modal plasticity compared to late blind individuals.

## Teaser

Bang *et al*. reported that the nucleus basalis of Meynert, the major source of cholinergic signals, presents diverging patterns of plasticity across early- and late-onset blindness. While its directionality of water diffusion is reduced in both early- and late-onset blindness, its functional connectivity is increased only in early blind individuals.

## Graphical Abstract

**Graphical Abstract:**
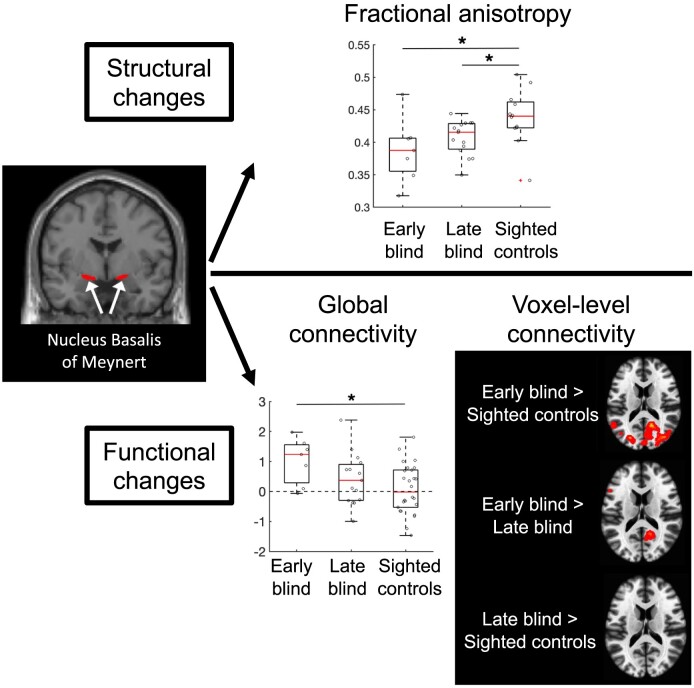

## Introduction

Loss of vision leads to compensatory use of the spared sensory modalities. An ample amount of evidence indicates that blind individuals perform better than sighted people in various non-visual tasks, including echolocation,^1,2^ pitch discrimination,^3^ speech discrimination,^4^ tactile discrimination^5,6^ and verbal memory.^7–9^ This superior ability of blind individuals is thought to be subserved by cross-modal plasticity, whereby cortical areas of the deprived modality are recruited by intact sensory modalities. Prior studies have demonstrated that blind individuals’ visual cortex becomes recruited for a wide range of non-visual tasks ranging from perceptual to cognitive tasks.^10,11^ Further, the visual cortex of blind individuals was shown to have greater functional connections with the brain areas associated with attention compared to sighted individuals.^12,13^

One of the critical questions about cross-modal plasticity points to how it arises from visual deprivation. A growing consensus is that cross-modal plasticity in blindness is shaped by the developmental sensitive period.^14–16^ While both early and late blind individuals present cross-modal recruitment of the visual cortex during non-visual tasks, its intensity and spatial extent are generally reduced with increasing age of blindness onset.^17–21^ Further, the visual cortex appears to be sensitive to task complexity only in early blind individuals. The visual cortex presents greater activity in response to complex sentences or mathematical equations over simple ones in early blind individuals but not in late blind individuals.^16,22^ This finding suggests that cross-modal plasticity may serve different functionalities during task performance. Indeed, when the function of the visual cortex was interrupted by stroke or transcranial magnetic stimulation, behavioural performance on the non-visual tasks was severely impaired in early blind individuals^23–26^ but not in late blind individuals.^23^ Similarly, early blind individuals present a tight correlation between the activity of the visual cortex and behavioural performance during non-visual tasks.^7,27,28^ However, such evidence is relatively sparse in late blind individuals. Prior studies also suggest that this discrepancy in the cross-modal plasticity between early- and late-onset blindness may be related to spontaneous brain activity during rest. While both early and late blind individuals present increased functional connectivity between occipital visual and frontotemporal language areas during rest,^29,30^ its degree of strength is weaker in late-onset blindness.^16^ This convergence of results suggests that the timing of visual deprivation plays a key role in cross-modal plasticity.

To date, one of the proposed but understudied mechanisms underlying cross-modal plasticity can be attributed to the cholinergic signals.^31^ The cholinergic nervous system takes part in attention^32,33^ and experience-dependent cortical plasticity.^34–36^ Among the major cholinergic pathways, the nucleus basalis of Meynert (NBM) located within the basal forebrain provides the principal source of cholinergic signals to the cortex. Prior experiments in monkeys and humans showed that the cholinergic neurons in the NBM innervate the cerebral cortex, including both primary sensory areas and high-order association areas.^37–39^ Physiologically, neuronal plasticity and sensory coding of the stimulus in cortical areas can be regulated by the cholinergic neurons in the NBM.^40^ For example, when a sensory input is presented concurrently with electrical stimulation of the NBM, the receptive field in the sensory cortex is shifted toward the paired stimulus^35^ and both the cortical representation of the stimulus^41–43^ and the behavioural performance are improved.^42,44^ In addition to local cortical modulation, the NBM contributes to coordinating the global pattern of brain activity. When either the left or right NBM is inactivated by local muscimol infusion, the global signal ipsilateral to the injection site is suppressed.^45^ This line of studies indicates that the NBM modulates cortical functions and behaviour, providing support for the notion that cross-modal plasticity may involve the NBM.

However, there is no direct evidence that the NBM is involved in cross-modal plasticity under visual deprivation. Considering that early blind individuals often present superior performance in non-visual tasks^3,21,46,47^ and greater intensity and spatial extent of cross-modal plasticity compared to late blind individuals, it is plausible that cholinergic modulation of the NBM may be stronger in early blind individuals. Specifically, we hypothesize that the NBM may undergo distinct developmental phases across early blind, late blind and sighted individuals, resulting in different structural and functional properties. At the structural level, the white matter microstructure of the NBM may be altered in early blind individuals because the synaptic pruning process is dependent on visual input during the developmental period.^48^ At the functional level, the connectivity between the NBM and cortical areas may be enhanced in early-onset blindness, given that early blind individuals present superior performance in various non-visual tasks while attention and sensory coding can be boosted by cholinergic signals originating from the NBM.^35,40–42,44^ Further, cerebrovascular reactivity, which refers to the ability of the blood vessels to dilate in order to match tissue blood supply to increased demand, may be enhanced within the NBM in early-onset blindness if cross-modal plasticity is accompanied by increased metabolic demands or neuronal activities of the NBM. To test our hypothesis encompassing both structure and function, we focused on the NBM’s volumetric size, white matter integrity, cerebrovascular reactivity and functional connectivity using multiparametric MRI. This examination would shed light on the mechanisms of plasticity and may further provide imaging biomarkers of cross-modal plasticity in blind individuals.

## Materials and methods

### Participants

Forty-nine subjects (29 females, mean ± SE age: 54.67 ± 2.12) without any history of neurological disorders participated in the study. Seven subjects were early blind (4 females, age: 55.43 ± 5.18, age of blindness onset: 0), 16 subjects were late blind individuals (8 females, age: 53.38 ± 3.81, age of blindness onset: 38.25 ± 4.32), and 26 subjects were sighted controls (17 females, age: 55.27 ± 3.02). All subjects were scanned inside a 3-tesla Siemens Allegra magnetic resonance (MR) scanner for anatomical and functional MR imaging. Seven early blind, 16 late blind and 13 sighted individuals also underwent diffusion tensor imaging (DTI) for assessing microstructural brain integrity. The demographic data of the early and late blind individuals are depicted in Supplementary Table 1. Age and gender were comparable across the three groups [age: *F*(2,46) = 0.088, *P* = 0.916, partial *η*^2^ = 0.004, one-way ANOVA; gender: *χ*^2^(2) = 0.985, *P* = 0.611, Phi = 0.142, Pearson chi-square test]. This study was approved by the Institutional Review Board of the University of Pittsburgh. All subjects provided written informed consent.

### MRI data acquisition

Anatomical MR images were obtained using a 3D T_1_-weighted magnetization-prepared rapid acquisition with gradient echo (MPRAGE) sequence, with 176 contiguous 1 mm sagittal slices, voxel size = 1 × 1 × 1 mm^3^, repetition time (TR) = 1400 ms, echo time (TE) = 2.5 ms, flip angle = 8°, field of view (FOV) = 256 × 256 mm^2^ and acquisition matrix = 256 × 256. DTI data were obtained using a spin–echo diffusion-weighted echo-planar imaging (EPI) sequence, with 36 contiguous 2.5 mm axial slices, 50 diffusion gradient directions at diffusion weighting factor (*b*) = 1200 s/mm^2^ and 5 non-diffusion-weighted *b*_0_ images at *b* = 0 s/mm^2^, TR = 5400 ms, TE = 112 ms, FOV = 240 × 240 mm^2^ and acquisition matrix = 96 × 96. Functional images were obtained for 8 min using a single-shot gradient-echo EPI sequence, with 36 contiguous 3.2 mm axial slices, voxel size = 3.2 × 3.2 × 3.2 mm^3^, TR = 2000 ms, TE = 25 ms, FOV = 205 × 205 mm^2^, acquisition matrix = 64 × 64 and scanning volume = 240. During the acquisition of functional images, subjects were instructed to close their eyes but stay awake. The slices covered the whole brain. One late blind individual was later excluded from the functional connectivity and cerebrovascular reactivity analyses because the functional MRI scan failed to cover the NBM and other basal forebrain regions. Additionally, we excluded one early blind individual from the cerebrovascular reactivity analysis and one late blind individual and one sighted individual from the DTI analysis due to technical issues.

### Seed regions of interest

The anatomical maps of the NBM and the remaining parts of the basal forebrain were obtained from post-mortem human brains in a recent study.^49^ Specifically, the map of ‘cholinergic cell group 4 (Ch4)’ corresponding to the NBM and the map of remaining parts of the basal forebrain, that is ‘cholinergic cell groups 1–3 (Ch1–3)’ were defined from stereotaxic maps using the SPM Anatomy toolbox version 3.0.^50^ Here, Ch1 refers to the medial septum, Ch2 refers to the vertical limb of the diagonal band, and Ch3 refers to the horizontal limb of the diagonal band. Ch1–3 were segmented together in the SPM Anatomy toolbox due to their close proximity. We used these Ch4 and Ch1–3 maps throughout the entire analysis. For DTI analysis, Ch4 and Ch1–3 maps had to be resampled to match the space of DTI using the Functional Magnetic Resonance Imaging of the Brain (FMRIB) Software Library (FSL) (http://www.fmrib.ox.ac.uk/fsl).

### Voxel-based morphometry analysis

We conducted voxel-based morphometry (VBM) analysis on the anatomical MRI data to test whether the NBM presents any volumetric changes within the grey and white matter of the NBM. Following the default pipeline of the Computational Anatomy Toolbox (CAT12) running under Statistical Parametric Mapping (SPM12) (http://www.fil.ion.ucl.ac.uk/spm/), the 3D T_1_-weighted MR images were registered into the stereotactic space using an affine transformation and non-linear registration and were segmented into grey matter tissues, white matter tissues and cerebrospinal fluid. Then, the segmented images were normalized to the Montreal Neurological Institute (MNI) space using the Diffeomorphic Anatomical Registration Through Exponentiated Lie Algebra (DARTEL) software. These images were then smoothed using a Gaussian kernel of 6 mm full width at half maximum (FWHM). We extracted the volumes of the grey matter and white matter within the NBM and Ch1–3 using a default segmentation pipeline in CAT12. Each individual’s total intracranial volume was obtained by summing up the total grey matter, white matter and cerebrospinal fluid volumes.

### DTI tract-based spatial statistics

We used tract-based spatial statistics (TBSS)^51^ in the FSL to investigate changes in the white matter integrity of the NBM. The diffusion-weighted images were first corrected for eddy currents and head motion. Then, we obtained the fractional anisotropy (FA) images using TBSS and aligned the images into the MNI template using the non-linear registration function in FSL. Next, we averaged the FA images to obtain a group mean FA map. This group mean FA map was then used to obtain a mean FA skeleton of the white matter tracts. We projected each individual’s FA images onto the mean FA skeleton. The voxel-wise statistical analysis was performed using the non-parametric permutation method in FSL (‘randomize’ function). We tested significance using the threshold-free cluster enhancement with family-wise error (FWE) correction. The FA values of the NBM as well as those of Ch1–3 and optic radiation were extracted from the voxels on the skeleton space for quantitative evaluation.

### Relative cerebrovascular reactivity analysis

Cerebrovascular reactivity is a measure of the cerebral blood vessels’ ability to dilate or constrict in response to vasoactive stimuli.^52^ Enhanced cerebrovascular reactivity implies increased blood delivery to brain regions that have enhanced metabolic demands and neural activities. This cerebrovascular reactivity is typically measured using hypercapnic gas inhalation as a vasoactive challenge.^53^ However, recently developed new technology allows us to obtain the relative cerebrovascular reactivity (rCVR) maps from resting-state functional MRI without gas inhalation.^54,55^ Thus, here we obtained the rCVR maps on the MNI space from the resting-state functional MRI images using the below formulae available at https://mricloud.org/.^56^


(1) $$\frac{\Delta \mathrm{BOLD}}{\mathrm{BOLD}}=\alpha ∙GS+\beta$$

(2) $$r\mathrm{CVR}=\frac{\alpha }{\mathrm{SI}}$$

In brief, voxel-wise CVR index (*α*) was computed using a general linear model between the normalized blood-oxygenation-level-dependent (BOLD) time series (ΔBOLD/BOLD) and the global signal (GS) time series that represented the BOLD time series of the whole brain. The voxel-wise rCVR values were then calculated by normalizing *α* by tissue signal intensity (SI) that was averaged across the whole brain. The residuals term (*β*) contained signals outside of the global signal, such as imaging noise, physiological noise, etc., and was not used for the analysis. Lastly, we extracted the rCVR values within specific regions, including the NBM and Ch1–3.

### Resting-state functional connectivity analysis

Resting-state functional MRI images were preprocessed using the functional connectivity toolbox for correlated and anticorrelated brain networks (CONN) version 18.a (www.nitrc.org/projects/conn,RRID:SCR_009550).^57^ The default preprocessing steps included realignment, unwarping, slice timing correction, segmentation, normalization into MNI space and smoothing using a Gaussian kernel of 8 mm FWHM. After preprocessing, images were band-pass filtered to 0.008–0.09 Hz and denoised using an anatomical component-based noise correction (aCompCor) procedure implemented in the CONN toolbox. This aCompCor procedure removed the noise components from cerebral white matter and cerebrospinal fluid and estimated subject-motion parameters from the functional images for each voxel and each subject.

Next, we performed the functional connectivity analyses (i.e. global correlation analysis, voxel-level correlation analysis and network-level correlation analysis) using the CONN toolbox. For global correlation analysis, we calculated the correlation coefficients between each voxel and the rest of the brain voxels across time series. These correlation coefficients were averaged across time series per voxel. Then, we extracted the global correlation coefficients from the voxels corresponding to the bilateral NBM (or bilateral Ch1–3) and averaged them to identify its brain-wide correlation properties.

For voxel-level analysis, we obtained the temporal correlation coefficients between the bilateral NBM (or bilateral Ch1–3) and each brain voxel. Then, we converted them to *z*-values using Fisher’s *r*-to-*z* transformation. Additionally, we performed the network-level analysis for cross-validation. CONN provides 30 cortical networks, including four default mode networks (bilateral lateral parietal cortex, medial prefrontal cortex, posterior cingulate cortex), four dorsal attention networks (bilateral frontal eye fields, bilateral intraparietal sulcus), four frontoparietal networks (bilateral lateral prefrontal cortex, bilateral posterior parietal cortex), four language networks (bilateral inferior frontal gyrus, bilateral posterior superior temporal gyrus), seven salience networks (anterior cingulate cortex, bilateral anterior insular cortex, bilateral rostral prefrontal cortex, bilateral supramarginal gyrus), three sensorimotor networks (bilateral lateral sensorimotor cortex, superior sensorimotor cortex) and four visual networks (bilateral lateral visual cortex, medial visual cortex, occipital visual cortex). These networks were derived from a healthy control dataset (497 subjects) using independent component analyses.^57^ Among 30 networks, a total of seven were our networks of interest because they spatially overlapped with the clusters of voxels that showed significant group differences from the voxel-level analysis. These were four visual networks (bilateral lateral visual cortex, medial visual cortex, occipital visual cortex), two language networks (left inferior frontal gyrus, left posterior superior temporal gyrus) and one default mode network (posterior cingulate cortex). We computed the temporal correlation coefficients between the bilateral NBM (or bilateral Ch1–3) and each of the cortical networks and converted them to *z*-values using Fisher’s *r*-to-*z* transformation.

### Statistical analysis

For whole-brain TBSS analysis, we used non-parametric permutation-based inference with statistical significance set at *P* < 0.05, corrected for multiple comparisons via the FWE method. For whole-brain voxel-level analysis of the functional connectivity, we used a voxel-wise height threshold of *P* < 0.001 and a cluster height threshold of false discovery rate (FDR)-corrected *P* < 0.05. For all other statistical analyses, we used two-tailed parametric tests with statistical significance at *P* < 0.05, corrected for multiple comparisons via the Holm–Bonferroni or Bonferroni methods. We reported the corrected *P*-values in all analyses. We assessed the assumption of sphericity using Mauchly’s sphericity tests and reported the Greenhouse–Geisser corrected results when the assumption of sphericity was violated. We also tested the assumption of homogeneity of variance using Levene’s test. When Levene’s test was significant, we reported the Welch and Games–Howell test results. For general linear regression models, we included the early and late blind individuals only and predicted the functional connectivity of the NBM from the age at onset of blindness while controlling for the years of age. Before conducting general linear regression analyses, we assessed the assumptions of independence of observations and non-multicollinearity using the Durbin–Watson statistic and tolerance/VIF values. For statistical testing, we used IBM Statistical Package for Social Sciences (SPSS) Statistics version 25.0, R version 4.1.3 and built-in programs in FSL version 6.0.1 as well as CONN toolbox version 18.a.

## Results

Here, we present the multiparametric MRI results of the NBM’s structural volume, white matter microstructural integrity, cerebrovascular reserve and resting-state functional connectivity as follows.

### Effects of visual deprivation on the structure of the NBM

**Figure 1:**
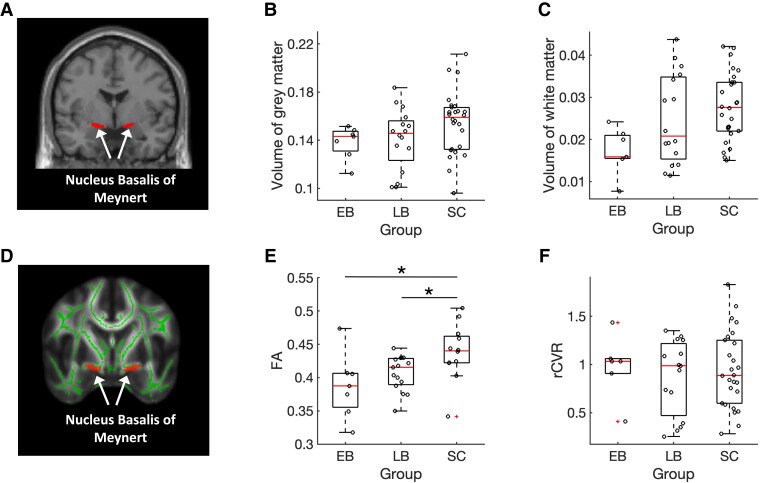
**Structure and cerebrovascular reactivity in the nucleus basalis of Meynert of blind individuals.** (**A**) Coronal view of the nucleus basalis of Meynert (arrow) in T_1_-weighted MRI. (**B**) The grey matter volume of the nucleus basalis of Meynert was comparable between early blind (EB), late blind (LB) and sighted control (SC) groups (*P* > 0.05). (**C**) The white matter volume of the nucleus basalis of Meynert did not differ between groups (*P* > 0.05). (**D**) Coronal view of the nucleus basalis of Meynert (arrow) overlaid on the mean FA skeleton (highlighted). (**E**) Mean FA within the nucleus basalis of Meynert is reduced in the early blind and late blind individuals compared to sighted controls [early blind versus sighted controls, *T*(29) = −3.009, Holm–Bonferroni *P* = 0.016; late blind versus sighted controls, *T*(29) = −2.408, Holm–Bonferroni *P* = 0.045]. (**F**) The rCVR of the nucleus Basalis of Meynert was comparable between groups (*P* > 0.05). The distributions are represented using box plots, and the outliers are plotted as plus signs. *Holm–Bonferroni corrected *P* < 0.05. For the volume of the grey and white matter, early blind: *n* = 7, late blind: *n* = 16, sighted controls: *n* = 26. For FA, early blind: *n* = 7, late blind: *n* = 15, sighted controls: *n* = 12. For rCVR, early blind: *n* = 6, late blind: *n* = 15, sighted controls: *n* = 26.

To investigate whether the structure of the NBM (Fig. 1A) was affected by blindness, we obtained the volume of the grey and white matter of the NBM and performed one-way ANCOVAs with a factor group (sighted control, early blind, late blind) while controlling for total intracranial volume and age. The results revealed no main effect of group for grey and white matter volumes [grey matter volume: *F*(2,44) = 1.510, *P* = 0.232, partial *η*^2^ = 0.064; white matter volume: *F*(2,44) = 1.664, *P* = 0.201, partial *η*^2^ = 0.070; Fig. 1B and C]. Whole-brain volumetric analysis also yielded no group difference in any of the voxels (FWE-corrected *P* > 0.05). Next, we investigated the white matter integrity within the NBM by examining the directionality of water diffusion using FA in DTI. We extracted the mean FA skeleton values from the NBM map and performed a one-way ANCOVA with a factor of group while controlling for total intracranial volume and age. The results showed that both early and late blind individuals had lower FA in the white matter of the NBM compared to sighted controls [main effect of group, *F*(2,29) = 4.948, *P* = 0.014, partial *η*^2^ = 0.254; early blind versus sighted controls, *T*(29) = −3.009, Holm–Bonferroni *P* = 0.016, late blind versus sighted controls, *T*(29) = −2.408, Holm–Bonferroni *P* = 0.045, early blind versus late blind, *T*(29) = −1.376, Holm–Bonferroni *P* = 0.179; Fig. 1E]. However, this FA difference did not survive the multiple comparison correction in the whole-brain analysis. Instead, the whole-brain analysis showed a reduction of FA in the bilateral optic radiations of blind individuals, which is consistent with prior studies^58–62^ (Supplementary Fig. 1).

### Null effects of visual deprivation on the cerebrovascular reactivity of the NBM

Next, we examined whether the NBM’s cerebrovascular reactivity was affected by blindness. For this, we conducted a one-way ANCOVA with a factor group (early blind, late blind, sighted controls) while controlling for age to the rCVR of the NBM. The results revealed no main effect of group in the NBM [*F*(2,43) = 0.257, *P* = 0.775, partial *η*^2^ = 0.012; Fig. 1F], suggesting that the cerebrovascular response was comparable across groups. While the rCVR of the NBM remained unchanged, the whole-brain analysis showed that rCVR may be lower in some of the cortical networks of blind individuals (for details, see Supplementary Figs 2 and 3).

### Effects of visual deprivation on the global-level functional connectivity of the NBM

**Figure 2:**
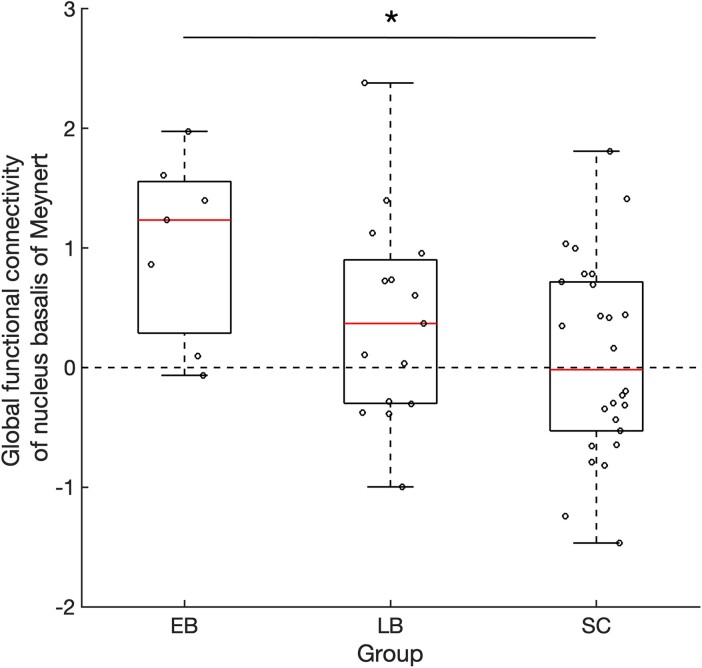
**Global functional connectivity of the nucleus basalis of Meynert.** Global functional connectivity between the nucleus basalis of Meynert and the entire cortical areas is increased in the early blind (EB) group compared to sighted controls (SC) [*T*(44) = 2.689, Holm–Bonferroni *P* = 0.030]. The distributions are represented using box plots. ‘LB’ refers to the late blind. *Holm–Bonferroni corrected *P* < 0.05. Each point represents one subject. Early blind: *n* = 7, late blind: *n* = 15, sighted controls: *n* = 26.

To investigate whether blindness alters the global connectivity of the NBM, we computed the functional connectivity between the NBM and all cortical voxels. We applied a one-way ANCOVA with a factor group (early blind, late blind, sighted controls) while controlling for age. The results showed a significant main effect of group [*F*(2,44) = 3.799, *P* = 0.030, partial *η*^2^ = 0.147; Fig. 2]. *Post hoc* tests showed that the early blind group had greater global connectivity compared to sighted controls (early blind versus sighted controls, *T*(44) = 2.689, Holm–Bonferroni *P* = 0.030). However, this increased global connectivity of the early blind group did not differ from that of the late blind group (early blind versus late blind, *T*(44) = 1.556, Holm–Bonferroni *P* = 0.254; late blind versus sighted controls, *T*(44) = 1.329, Holm–Bonferroni *P* = 0.254). In addition, we observed a decreasing linear trend among the three groups, suggesting that the global functional connectivity declined across early blind, late blind and sighted individuals [*F*(1,45) = 7.078, *P* = 0.011]. In line with this observation, a general linear regression analysis revealed that among early and late blind individuals, the age at onset of blindness predicted the global connectivity of the NBM while controlling for age [*T*(19) = −2.503, *P* = 0.022, *β* = −0.511]. This result indicates that the earlier one becomes blind in their life, the greater the global connectivity of the NBM becomes.

### Effects of visual deprivation on the voxel- and network-level functional connectivity of the NBM

**Figure 3:**
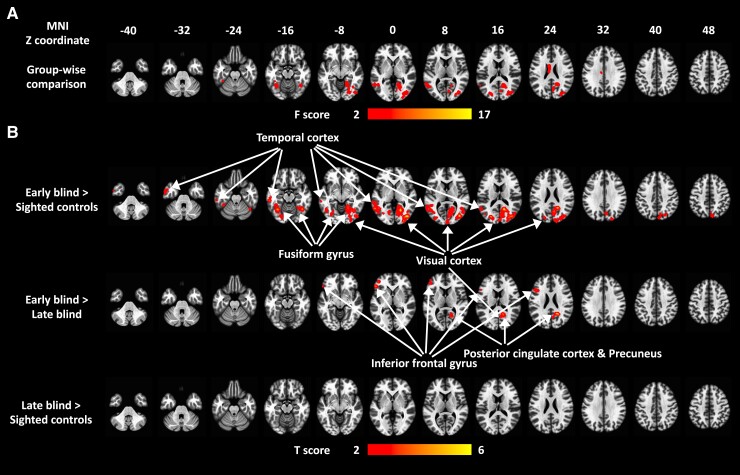
**Group differences in functional connectivity at the voxel level.** (**A**) *F* map for the group-wise comparisons across early blind, late blind and sighted controls. Significant group difference was observed in the bilateral visual cortex, bilateral fusiform area, right posterior cingulate cortex, right precuneus, left temporal gyrus and left angular gyrus. (**B**) *Post hoc t*-tests between groups. Compared to the sighted controls, the early blind individuals presented increased functional connectivity of the nucleus basalis of Meynert with the bilateral visual cortex, bilateral fusiform area, right posterior cingulate cortex, right precuneus, left temporal gyrus and left angular gyrus. Further, compared to the late blind individuals, the early blind group exhibited greater connectivity of the nucleus basalis of Meynert with the right posterior cingulate cortex, right precuneus, right visual cortex and left inferior frontal gyrus. The late blind and sighted individuals did not show any difference. Early blind: *n* = 7, late blind: *n* = 15, sighted controls: *n* = 26.

Having confirmed the existence of greater global connectivity of the NBM in early-onset blindness, we further examined whether similar patterns existed at the voxel level. For each cortical voxel, we computed its functional connectivity with the NBM. Then, we examined which cortical voxels presented altered functional connectivity with the NBM across groups. Results showed significant group differences within the bilateral visual cortex, bilateral fusiform area, right posterior cingulate cortex, right precuneus, left temporal gyrus and left angular gyrus (Fig. 3). *Post hoc* tests revealed that this group difference was driven by the increased connectivity in the early blind group (Table 1). Specifically, the NBM showed greater connectivity with the bilateral visual cortex (striate and extrastriate cortices), bilateral fusiform cortex, right posterior cingulate cortex, right precuneus, left temporal gyrus and left angular gyrus in the early blind individuals compared to sighted controls. Comparison of the early blind and late blind groups revealed that the NBM had greater connectivity with the right posterior cingulate cortex, right precuneus, right visual cortex (striate cortex) and left inferior frontal gyrus in the early blind compared to the late blind individuals. However, when comparing the connectivity between the late blind group and sighted controls, we observed no difference. These observations suggest that the NBM develops greater functional connectivity at a local level in early-onset blindness.

**Table 1:** Brain regions showing differences in functional connectivity of the nucleus basalis of Meynert between early blind individuals and sighted controls and between early blind and late blind individuals

Contrast	Hemisphere	Regions	Peak MNI coordinate			*t*-statistic	Cluster size	Size p-FDR
			*x*	*y*	*z*			
Early blind > sighted controls	Right	Striate & extrastriate cortex (BA 17, 18, 19) Posterior cingulate cortex (BA 23) Precuneus (BA 7) Fusiform cortex (BA 37)	38	−78	4	6.57	4280	<0.001
	Left	Angular gyrus (BA 39) Superior/middle/inferior temporal gyrus (BA 20, 21, 22) Fusiform cortex (BA 37)	−58	−58	12	6.96	1558	<0.001
	Left	Extrastriate cortex (BA 18, 19)	−28	−80	14	5.19	230	0.002
	Left	Middle/inferior temporal gyrus (BA 20, 21)	−58	−6	−36	4.93	124	0.038
	Left	Striate cortex (BA 17)	−16	−68	4	4.45	113	0.044
Early blind > late blind	Right	Posterior cingulate cortex (BA 23) Precuneus (BA 7) Striate cortex (BA 17)	22	−54	24	5.12	351	<0.001
	Left	Inferior frontal gyrus (pars triangularis, BA 45)	−54	30	0	6.05	215	0.004
	Left	Inferior frontal gyrus (pars opercularis, BA 44)	−44	18	24	4.68	129	0.034

**Figure 4:**
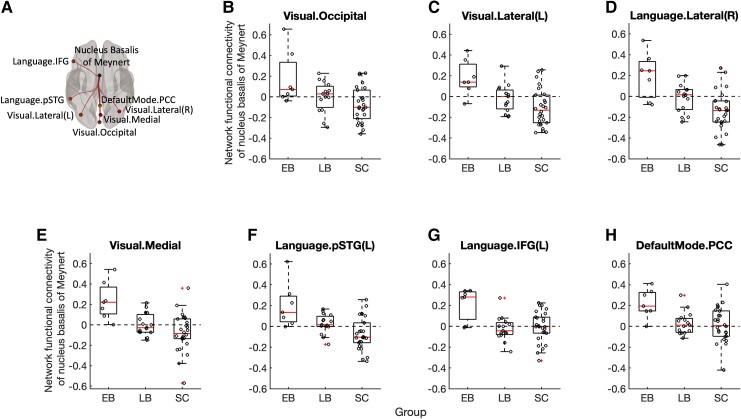
**Early blind individuals exhibit stronger functional connectivity between the nucleus basalis of Meynert and the visual, language and default mode cortical networks than late blind individuals and sighted controls.** (**A**) Schematic depiction of the nucleus basalis of Meynert and seven cortical networks (occipital, lateral, medial visual cortices, left posterior superior temporal gyrus, left inferior frontal gyrus, posterior cingulate cortex) that showed increased functional connectivity with the nucleus basalis of Meynert in the early blind group. (**B–H**) Functional connectivity between the nucleus basalis of Meynert and the seven cortical networks across early blind (EB), late blind (LB) and sighted control (SC) groups. The distributions are represented using box plots, and the outliers are plotted as plus signs. Early blind: *n* = 7, late blind: *n* = 15, sighted controls: *n* = 26.

Of note, cortical areas showing greater connectivity with the NBM in early-onset blindness spatially overlap with some of the large-scale intrinsic brain networks in the CONN toolbox, which were derived from the meta-analysis of resting-state fMRI studies.^57^ These include four visual networks (bilateral lateral visual cortex, medial visual cortex, occipital visual cortex), two language networks (left inferior frontal gyrus, left posterior superior temporal gyrus) and one default mode network (posterior cingulate cortex). Therefore, we further examined whether the increased connectivity of the NBM could also be captured from these seven networks. For each cortical network, we computed its functional connectivity with the NBM. Then, we conducted a two-way mixed-measures MANCOVA with factors group (early blind, late blind, sighted controls) and network (the seven networks) while controlling for age. The results revealed a significant main effect of group [*F*(2,44) = 15.608, *P* < 0.001, partial *η*^2^= 0.415], with no main effect of network [*F*(6,264) = 1.343, Greenhouse–Geisser correction, *ε*=0.726, *P* = 0.253, partial *η*^2^ = 0.030] or interaction between factors group and network [*F*(12,264) = 1.126, Greenhouse–Geisser correction, *ε*=0.726, *P* = 0.346, partial *η*^2^ = 0.049; Fig. 4]. *Post hoc* tests demonstrated that the functional connectivity of the early blind individuals was greater than that of the sighted controls or late blind individuals across all seven networks (early blind versus sighted controls: Bonferroni *P* < 0.001, 95% CI = 0.150–0.392; late blind versus sighted controls: Bonferroni *P* = 0.197, 95% CI = −0.022–0.162; early blind versus late blind: Bonferroni *P* = 0.001, 95% CI = 0.071–0.331). Other networks derived from the meta-analysis, however, did not present these changes (*P* > 0.05) (Supplementary Fig. 4).

Further, we observed a decreasing linear trend among the three groups in all seven networks, indicating that the connectivity declined across early blind, late blind and sighted individuals {visual networks [occipital visual cortex: *F*(1,45) = 10.256, *P* = 0.003; left lateral visual cortex: *F*(1,45) = 16.723, *P* < 0.001; right lateral visual cortex: *F*(1,45) = 21.153, *P* < 0.001; medial visual cortex: *F*(1,45) = 18.230, *P* < 0.001], language networks [left posterior superior temporal gyrus: *F*(1,45) = 17.955, *P* < 0.001; left inferior frontal gyrus: *F*(1,45) = 8.885, *P* = 0.005] and default mode network [posterior cingulate cortex: *F*(1,45) = 7.498, *P* = 0.009]}. Consistent with these observations, general linear regression analyses showed that the age at onset of blindness predicted the functional connectivity of the NBM after adjustment for age in all networks except the occipital visual cortex {visual networks [occipital visual cortex: *T*(19) = −1.964, *P* = 0.064, *β* = −0.423; left lateral visual cortex: *T*(19) = −3.186, *P* = 0.005, *β* = −0.604; right lateral visual cortex: *T*(19) = −2.866, *P* = 0.010, *β* = −0.574; medial visual cortex: *T*(19) = −3.608, *P* = 0.002, *β* = −0.668], language networks [left posterior superior temporal gyrus: *T*(19) = −2.409, *P* = 0.026, *β* = −0.495; left inferior frontal gyrus: *T*(19) = −2.472, *P* = 0.023, *β* = −0.522] and default mode network [posterior cingulate cortex: *T*(19) = −2.661, *P*= 0.015, *β* = −0.548]} among early and late blind individuals. These results show that the NBM develops greater functional connectivity with the visual, language and default mode networks as one becomes blind earlier in their life.

### Analyses of other basal forebrain regions

We also examined the potential effects of visual deprivation on the remaining parts of the basal forebrain (Ch1–3), which include the medial septal nucleus, the vertical limb of the diagonal band nucleus and the horizontal limb of the diagonal band nucleus (Supplementary Fig. 5A). Our analysis did not yield any changes in Ch1–3’s grey or white matter volume [grey matter volume: *F*(2,44) = 0.873, *P* = 0.425, partial *η*^2^ = 0.038; white matter volume: *F*(2,44) = 0.863, *P* = 0.429, partial *η*^2^ = 0.038; Supplementary Fig. 5B and C], white matter microstructural integrity [*F*(2,43) = 1.436, *P* = 0.249, partial *η*^2^ = 0.063; Supplementary Fig. 5D and E], rCVR [*F*(2,42) = 1.076, *P* = 0.350, partial *η*^2^ = 0.049; Supplementary Fig. 5F] or functional connectivity at either a global [*F*(2,44) = 0.636, *P* = 0.534, partial *η*^2^ = 0.028; Supplementary Fig. 5G] or a voxel (all *P*s > 0.05) or a network level (all *P*s > 0.05; Supplementary Fig. 6) across groups. These results suggest that the structural and functional changes that we observed in the NBM are not generalized to the remaining parts of the basal forebrain.

## Discussion

We examined whether the age at onset of blindness impacts the NBM’s structural and functional properties. The results demonstrated that the NBM’s volumetric size and cerebrovascular reactivity remained intact under visual deprivation but that its FA value in DTI was reduced to a similar extent across both early and late blind individuals. Notably, the functional connectivity of the NBM was observed to diverge between early and late blind individuals. The early blind individuals presented strengthened functional connectivity at both global and local levels, whereas the late blind individuals showed a little-to-no increase in functional connectivity at either a global or a local level compared to sighted controls. The cortical areas that showed increased functional connectivity with the NBM in the early blind compared to late blind individuals included the cortices that are dedicated for visual and language processing as well as a central node of the default mode network. All of these areas are known to receive cholinergic projections from the NBM.^37,63^ Furthermore, the age at onset of blindness predicted both global and local connectivity. These results suggest that upon reduced directionality of water diffusion, the NBM may exert greater cholinergic influences on the cortex of early blind individuals compared to late blind individuals.

As >90% of the neurons in the NBM are cholinergic,^37^ our finding of increased functional connectivity of the NBM in the early blind suggests that these individuals may be receptive to greater cholinergic input. The cholinergic neurons in the NBM are known to modulate the cortical processes via their dense projections to the cortex. Such cholinergic innervations can play a key role in attention,^32,33^ experience-dependent plasticity^34–36^ and sensory processing^41,42^ via cortical disinhibition.^40^ Stimulation of the NBM leads to a reduction of synaptic inhibition but an increase in excitation in the cortex.^35,64^ For example, repetitive pairing of the NBM stimulation with an external stimulus shifts the tuning curve of the cortical neurons toward the paired stimulus by inducing a decrease in synaptic inhibition but an increase in synaptic excitation.^35^ After exposure to the stimulus, reduced synaptic inhibition gradually increases until it balances the increased synaptic excitation.^35^ This cortical disinhibition during a restricted time was thus suggested to facilitate sensory processing in a stimulus-specific manner^40^ and to underlie mechanisms of plasticity. Further, a study showed that the timescale of the NBM’s modulation can be as short as subsecond to seconds.^42^ For example, when the cholinergic neurons in the NBM are activated using optogenetic tools, V1 responses increase at the scale of seconds and the behavioural performance is improved as well.^42^ A positive correlation was also found between the release of choline in the cortex and behavioural performance.^65^ These lines of studies suggest that under the condition where the cholinergic signal is enhanced, its impact on attention, sensory processing and experience-dependent plasticity could be further boosted.

In the current study, increased functional connectivity of the NBM was observed at both global and local levels in early blind individuals. This finding suggests enhanced cholinergic influence at two levels. At the global level, the NBM may exert greater influence on coordinating large-scale brain activity in early blind individuals.^45^ At the network level, cortical functions related to plasticity, attention and sensory processing may be enhanced within the visual, language and default mode networks of early blind individuals.^40^ Such enhancement of cortical functions then may facilitate superior behavioural performance. Indeed, early blind individuals perform better than sighted individuals^1,3–5,7^ and often better than late blind individuals^3,21,46,47^ during various non-visual tasks such as auditory, tactile and language tasks. Thus, it is speculated that there may be a functional link between enhanced cholinergic signals of the NBM and superior performance in early blind individuals. However, since the current study examined resting-state brain activities, this question needs to be further tested using task-based fMRI.

In contrast to early blind individuals, late blind individuals displayed a little-to-no increase in the functional connectivity of the NBM. This sharp contrast in the functional connectivity of the NBM between early- and late-onset blindness is likely affected by the critical or sensitive periods. Sensitive periods refer to the time window in development when the effects of experience on the brain are unusually strong compared to adulthood.^66^ Critical periods represent a special time window within the sensitive periods when experience induces irreversible alterations in the brain.^66^ While the visual functions in humans have their own timing of onset and emergence,^67^ it is thought that the sensitive periods for the visual functions end before puberty.^68^ Critically, the neurobiological mechanisms underlying brain development are known to be influenced by visual experience. For example, the amount of choline in the brain is known to peak at 3 months of age and then decline until it reaches a plateau by early childhood.^69,70^ However, when vision is deprived during this time window, this declining process of choline metabolism can be interrupted, resulting in a larger amount of choline. Prior studies using magnetic resonance spectroscopy (MRS) also demonstrated that early blind individuals present a greater amount of choline in the visual cortex compared to sighted controls.^31,71^ Although late blind individuals were not examined in the aforementioned studies, it is expected that those who become blind after early childhood are likely commensurate with the normal reduction in choline during early brain development,^69,70^ resulting in similar amounts of choline in the brain as the sighted individuals.

Another potential mechanism that may have contributed to the different connectivity of the NBM between early- and late-onset blindness is the synaptic pruning process. Similar to choline metabolism, the synaptic density of the visual cortex reaches its maximum during the first year and then gradually decreases up to ∼11 years of age through synaptic pruning.^72^ However, this process is interrupted under circumstances of early visual deprivation.^48^ As a result, the visual cortex is thicker in early-onset blindness compared to late-onset blindness.^73,74^ This disrupted pruning process is considered to enable more exuberant connectivity between distant brain regions in early-onset blindness.^75^ For example, the early blind but not sighted individuals manifest coupling between extrastriate visual regions and frontotemporal semantic regions during the semantic retrieval task.^75^ These studies proffer that in the case of late-onset blindness, the pruning process, having been completed before the onset of visual deprivation, prevents the NBM from developing compensatory changes in functional connectivity in spite of significant sensory deprivation. Thus, the enhanced sensory abilities of the early blind may be a function not of developing enhanced, new connections to recoup what has been lost but rather hijacking the neuronal superhighways that were not eliminated during critical periods of development.

The neuronal alterations resulting from interactions between critical/sensitive periods and the timing of blindness likely underlie the varying levels of cross-modal plasticity during non-visual tasks. Results of this study suggest that the strengthened functional connectivity of the NBM in early blind individuals is a possible candidate at play during cross-modal plasticity. In particular, the greater connectivity of the NBM in early- but not in late-onset blindness is consistent with the notion that cross-modal plasticity is generally reduced with increasing age of blindness onset.^17–21^ Further, prior studies demonstrated that early blind individuals present greater brain activity compared to sighted individuals within the occipital visual cortex,^7,17,76–79^ the fusiform area^15,80^ (which is a part of the visual network) and the left superior temporal sulcus^80,81^ (which is a part of the language network) during non-visual tasks. Consistent with these results, our data also showed that these cortical areas present enhanced functional connectivity with the NBM in early-onset blindness. Future work may elucidate whether the greater brain activity in these cortical areas has any associations with the increased functional connectivity of the NBM in early blind individuals.

In addition to the connectivity of the NBM, there are several other mechanisms that have been proposed to underlie cross-modal plasticity. These include changes in local cortical connectivity, subcortico-cortical connectivity and cortico-cortical feedback.^82^ It is an open question whether these mechanisms have any association with the NBM. Future studies using task-based fMRI are needed to directly examine this question.

Our study shows that the blind exhibit lower FA in the NBM. An important question that emerges is how to reconcile a lower FA in the NBM with strengthened functional connectivity in the early blind and little-to-no alterations in functional connectivity in the late blind. Lower FA can arise from a higher proportion of crossing fibres, reduction of intra- or interhemispheric fibres, reduced axonal maturation or increased demyelination. A hypothesis for the early blind (who show enhanced functional connectivity of the NBM) is that because the ample, nascent cholinergic projections are not properly eradicated during development, at baseline, this cohort possesses a greater number of cholinergic projections with varying directions and distribution of fibres. The NBM may exploit these highways to its advantage to develop greater cholinergic influence on global brain activity and local cortical activity.

However, in the case of late blind individuals, reduced FA is likely to have different underlying mechanisms. Notably, since the NBM of late blind individuals proceeds through the same normal developmental phases as sighted individuals, the structural consequence of this normal maturation process may culminate in unaffected functional connectivity of the NBM in spite of blindness. However, considering that visual deprivation provokes rapid structural changes,^83,84^ the reduced FA in the late blind may be a consequence of demyelination or reduction of intra- or interhemispheric fibres. To probe which structural property leads to reduced FA in early- and late-onset blindness, additional investigations using more advanced diffusion models are warranted. Furthermore, it should be noted that the volume and rCVR in the NBM did not change with visual deprivation. It suggests that the NBM may be physiologically intact in blind individuals regardless of age at onset of blindness.

The NBM is a complex structure with multiple subdivisions and subtypes of neurons. Although the NBM is dominated by cholinergic neurons, a subset of the NBM neurons is non-cholinergic (GABAergic or glutamatergic) and is involved in cortical arousal and salience encoding.^85–87^ According to tracer experiments on monkeys, the NBM consists of multiple subdivisions, each of which has its own preferred target on the cortical surface.^37^ For example, the anteromedial subdivision of the NBM projects to the medial cortex, including the cingulate cortex, the anterolateral region projects to the frontoparietal opercular cortex and the amygdala, the posterior region projects to the superior temporal and temporal polar areas, and the intermediate region projects to the remaining areas. In humans, the NBM was suggested to follow a similar pattern as in non-human primates.^63^ Considering the complexity of the NBM, it is speculated that the mechanisms of plasticity in the NBM may differ across its subdivisions and subtypes of neurons and that this difference may be impacted by the age at onset of blindness. These questions can be addressed in future studies using finer spatiotemporal resolution.

In the remaining parts of the basal forebrain (Ch1–3), no changes in the structural or functional properties were observed. This suggests that the plastic changes observed in the NBM are locally constrained and are not generalized to the remaining parts of the basal forebrain. This discrepancy between the NBM and Ch1–3 could be related to differences in the cortical projection pattern, functional connectivity profile or distribution of cholinergic neurons between these two regions.^63,88,89^

While the current study utilized multiple analytic methods to investigate changes in the NBM under visual deprivation, certain aspects were not covered in the study, such as cortical thickness and real-time cortical modulation of the NBM. Further, the sample size of the early blind individuals was small in the current study despite the statistical significance observed. Therefore, to validate our findings and to obtain a complete understanding of the basal forebrain, we need larger studies with more samples and various analytic tools in the future.

## Conclusion

To summarize, our results provide new insight into the plasticity of the NBM in visual deprivation. The NBM displayed preserved volume and cerebrovascular reactivity but a reduction in the directionality of water diffusion in early and late blind individuals. Notably, the NBM presented diverging patterns of functional connectivity between early and late blind individuals. The early blind individuals showed increased functional connectivity at both global and local levels, but the late blind individuals displayed connectivity similar to sighted controls. These results suggest that early blind individuals may be under stronger cholinergic modulation by the NBM. Building on these results, we propose that the differing levels of the NBM’s cholinergic influence may explain discrepancies in the cross-modal plasticity across early- and late-onset blindness and that the functional connectivity of the NBM may serve as a potential biomarker for cross-modal plasticity in blind individuals.

## Supplementary Material

fcad119_Supplementary_Data

## Data Availability

The global and network connectivity, rCVR, structural volume data and DTI FA data are freely available at https://osf.io/axy45/.

## Abbreviations

aCompCor =: anatomical component-based noise correction
BOLD =: blood-oxygenation-level-dependent
CAT12 =: Computational Anatomy Toolbox
Ch1–3 =: cholinergic cell groups 1–3
Ch4 =: cholinergic cell group 4
CONN =: functional connectivity toolbox for correlated and anticorrelated brain networks
DARTEL =: Diffeomorphic Anatomical Registration Through Exponentiated Lie Algebra
DTI =: diffusion tensor imaging
EPI =: echo-planar imaging
FA =: fractional anisotropy
FMRIB =: Functional Magnetic Resonance Imaging of the Brain
FOV =: field of view
FSL =: Functional Magnetic Resonance Imaging of the Brain Software Library
FWE =: family-wise error
FWHM =: full width at half maximum
MNI =: Montreal Neurological Institute
MR =: magnetic resonance
NBM =: nucleus basalis of Meynert
rCVR =: relative cerebrovascular reactivity
SI =: signal intensity
SPM12 =: Statistical Parametric Mapping
SPSS =: Statistical Package for Social Sciences
TBSS =: tract-based spatial statistics
TE =: echo time
TR =: repetition time
VBM =: voxel-based morphometry
