# Supplementary material for: Diverging patterns of plasticity in the nucleus basalis of Meynert in early- and late-onset blindness
Source: Brain Commun. 2023 Apr 11;5(2):fcad119. doi: 10.1093/braincomms/fcad119 (PMC10123399; doi:10.1093/braincomms/fcad119)
Supplement: fcad119_Supplementary_Data [file fcad119_supplementary_data.pdf]

Supplementary material

Gender	Age (years)	Age at onset of blindness (years)	Duration of blindness (years)	Cause of blindness	VBM analysis	DTI FA analysis	rCVR analysis	Functional connectivity analysis
M	25	0	25	congenital	Y	Y	Y	Y
M	56	0	56	congenital	Y	Y	Y	Y
F	58	0	58	congenital	Y	Y	Y	Y
F	60	0	60	retinopathy of prematurity	Y	Y	Y	Y
F	62	0	62	congenital	Y	Y	Y	Y
M	63	0	63	retinopathy of prematurity	Y	Y	N	Y
F	64	0	64	congenital	Y	Y	Y	Y
M	18	13	5	retinitis pigmentosa	Y	Y	Y	Y
F	30	23	7	tumors	Y	Y	Y	Y
F	35	31	4	ocular trauma	Y	Y	Y	Y
F	39	17	22	retinopathy of prematurity	Y	Y	Y	Y
F	53	28	25	diabetic retinopathy	Y	Y	Y	Y
M	55	35	20	ocular trauma	Y	Y	Y	Y
M	58	51	7	ocular trauma	Y	N	Y	Y
M	58	7	51	encephalitis	Y	Y	Y	Y
F	58	46	12	glaucoma	Y	Y	Y	Y
F	59	53	6	congenital cataracts, aniridia, pediatric glaucoma	Y	Y	Y	Y
M	59	54	5	post-surgery	Y	Y	N	N
F	60	31	29	glaucoma	Y	Y	Y	Y
M	62	51	11	ocular trauma	Y	Y	Y	Y
M	64	54	10	detached retinas	Y	Y	Y	Y
F	71	59	12	glaucoma	Y	Y	Y	Y
M	75	59	16	retinitis pigmentosa	Y	Y	Y	Y
M	21	NA	0	NA (sighted)	Y	Y	Y	Y

F	25	NA	0	NA (sighted)	Y	Y	Y	Y
M	27	NA	0	NA (sighted)	Y	Y	Y	Y
F	38	NA	0	NA (sighted)	Y	N	Y	Y
F	41	NA	0	NA (sighted)	Y	Y	Y	Y
F	44	NA	0	NA (sighted)	Y	Y	Y	Y
F	47	NA	0	NA (sighted)	Y	N	Y	Y
F	52	NA	0	NA (sighted)	Y	N	Y	Y
F	54	NA	0	NA (sighted)	Y	N	Y	Y
F	55	NA	0	NA (sighted)	Y	Y	Y	Y
F	55	NA	0	NA (sighted)	Y	N	Y	Y
F	55	NA	0	NA (sighted)	Y	N	Y	Y
M	56	NA	0	NA (sighted)	Y	Y	Y	Y
F	57	NA	0	NA (sighted)	Y	Y	Y	Y
F	58	NA	0	NA (sighted)	Y	Y	Y	Y
M	58	NA	0	NA (sighted)	Y	N	Y	Y
M	61	NA	0	NA (sighted)	Y	Y	Y	Y
M	62	NA	0	NA (sighted)	Y	N	Y	Y
F	64	NA	0	NA (sighted)	Y	N	Y	Y
F	68	NA	0	NA (sighted)	Y	N	Y	Y
F	70	NA	0	NA (sighted)	Y	Y	Y	Y
F	70	NA	0	NA (sighted)	Y	N	Y	Y
M	71	NA	0	NA (sighted)	Y	N	Y	Y
M	74	NA	0	NA (sighted)	Y	Y	Y	Y
M	75	NA	0	NA (sighted)	Y	N	Y	Y
F	79	NA	0	NA (sighted)	Y	N	Y	Y

Supplementary Table 1. Demographic, clinical, and analytic information of blind and sighted individuals. The top row shows gender, age, age at onset of blindness, duration of blindness, cause of blindness, and analyses used in the study. The rows below illustrate each individual. For DTI scans, we obtained the data from thirteen among twenty-six sighted individuals. Y indicates that the individual was included in the analysis. N indicates that the individual was not included in the analysis due to technical issues or incomplete data. NA refers to not applicable.

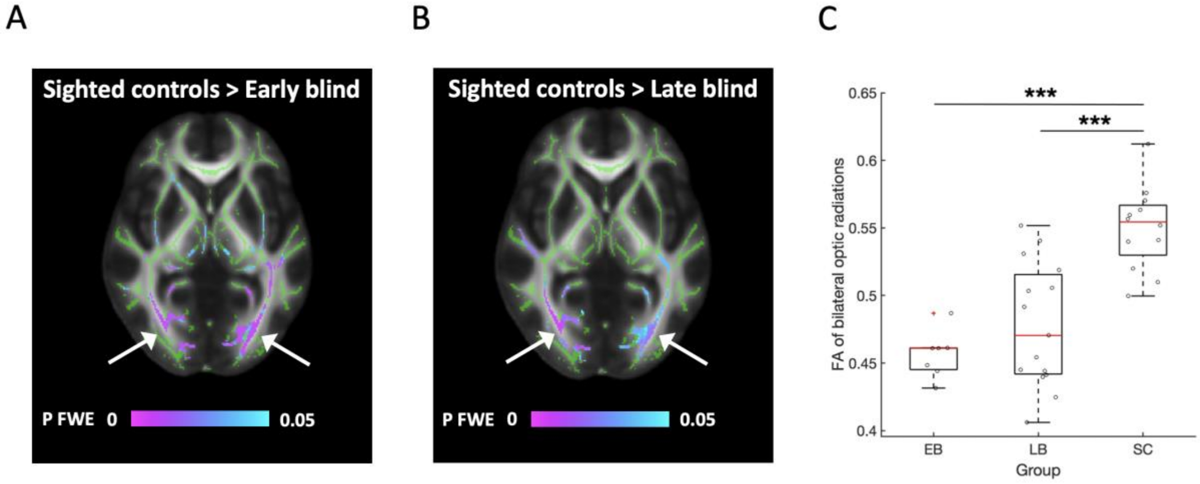

Supplementary Figure 1. Comparison of FA skeleton. (A-B) Statistical comparison of the whole brain FA skeleton indicates that both early blind (EB) and late blind (LB) individuals have reduced FA of the optic radiations (white arrows) compared to sighted controls (SC). Regions showing reduced FA (yellow-red) are overlaid on the mean FA skeleton (green). Threshold of P<0.05 after FWE correction was applied. (C) A region-of-interest analysis within the optic radiations showed the same results. A reduction of the mean FA was observed in both blind groups (bilateral optic radiations: $F(2,29)=16.407$, $P<0.001$, partial $\eta^2=0.531$, early blind vs. sighted controls, $T(29)=-4.990$, Holm-Bonferroni $P<0.001$, late blind vs. sighted controls, $T(29)=-5.075$, Holm-Bonferroni $P<0.001$, early blind vs. late blind, $T(29)=-1.339$, Holm-Bonferroni $P=0.191$; one-way ANCOVA with a factor group while controlling for total intracranial volume and age). The distributions are represented using box plots and the outliers are plotted as plus signs. ***Holm-Bonferroni corrected $P<0.001$. Early blind: $n=7$, late blind: $n=15$, sighted controls: $n=12$.

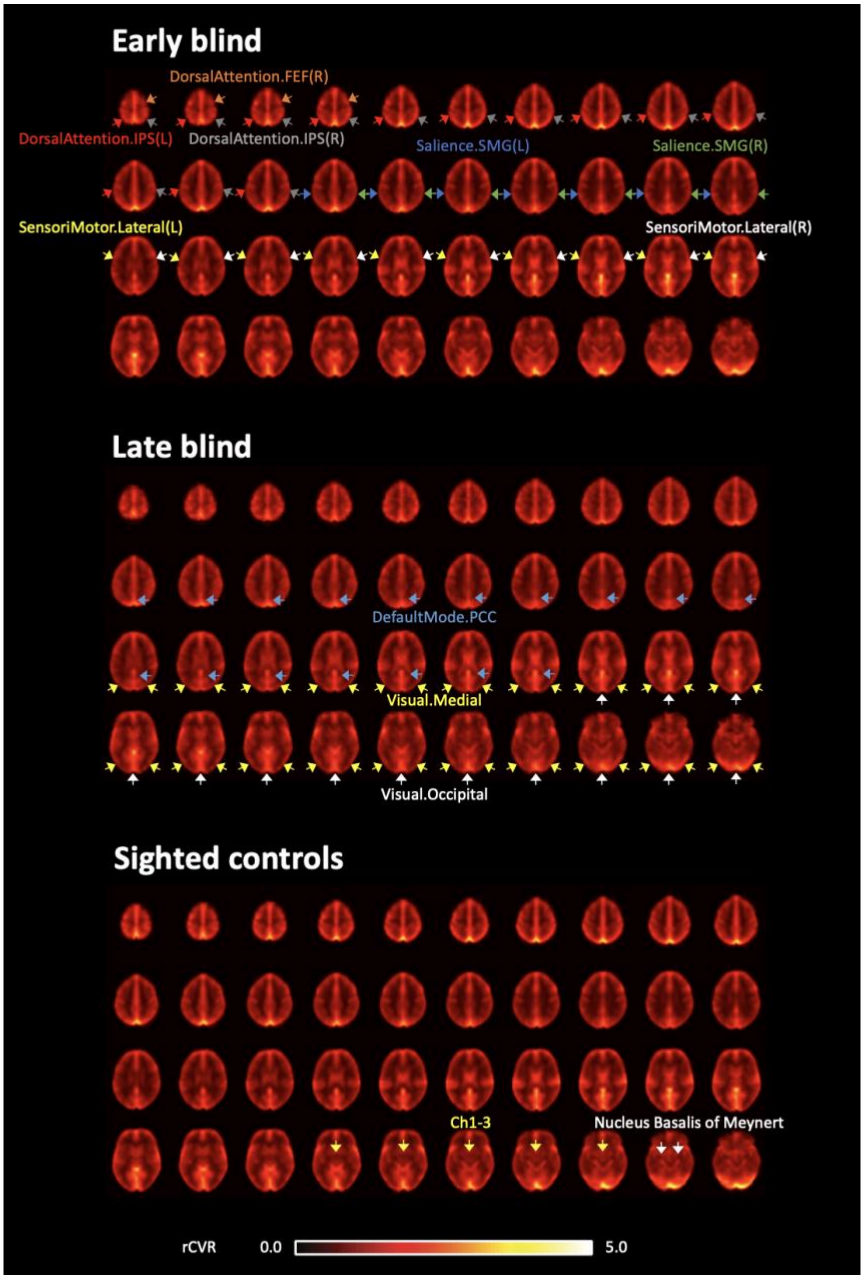

Supplementary Figure 2. Average rCVR maps for early blind, late blind individuals, and sighted controls across the whole brain. rCVR values (in arbitrary unit) for NBM and Ch1-3 appear comparable across groups. Early blind: *n*=6, late blind: *n*=15, sighted controls: *n*=26.

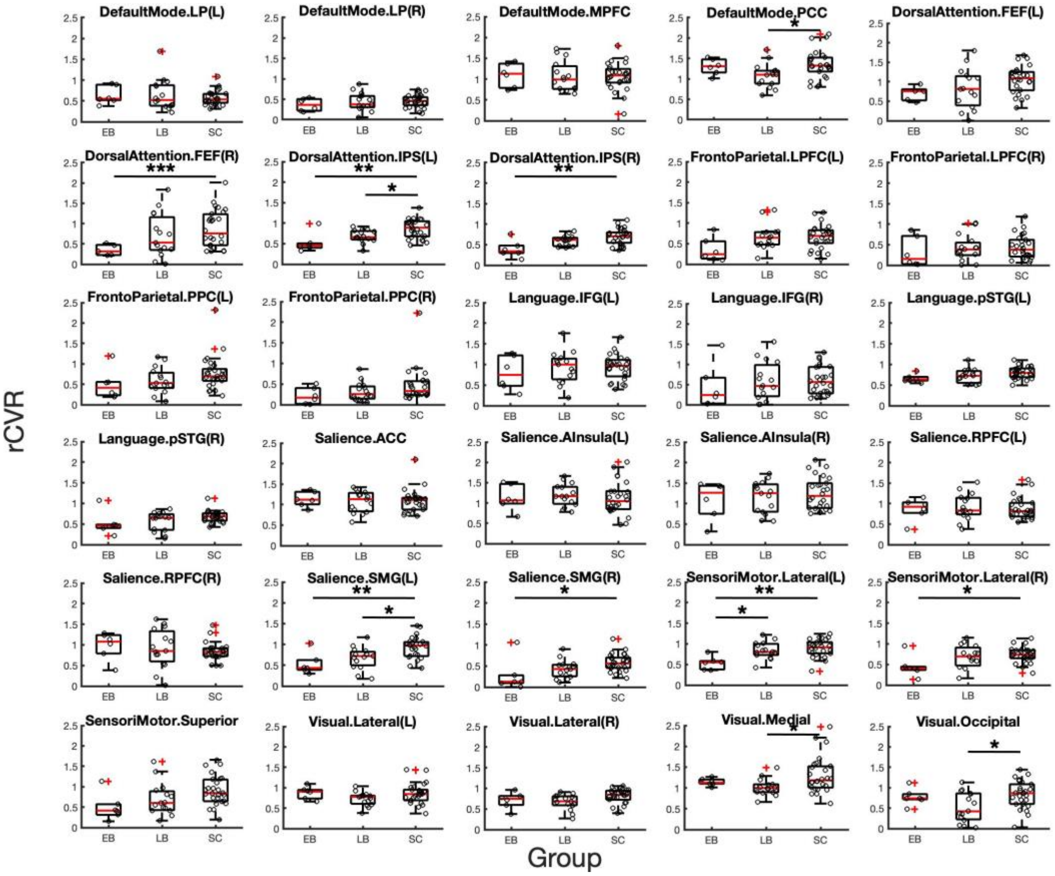

Supplementary Figure 3. rCVR of the thirty cortical networks. One-way ANOVAs with a factor group indicated reduced rCVR in the early blind (EB) individuals compared to sighted controls (SC) in the right frontal eye fields (main effect of group, $F_{welch}(2,23.238)=12.343$, $P<0.001$, partial $\eta^2=0.116$; early blind vs. sighted controls, Games-Howell $P<0.001$, 95% CI=-0.801 – -0.257), the bilateral intraparietal sulcus (left: main effect of group, $F(2,44)=8.499$, $P=0.001$, partial $\eta^2=0.279$; early blind vs. sighted controls, Bonferroni $P=0.002$, 95% CI=-0.582 – -0.111, late blind vs. sighted controls, Bonferroni $P=0.024$, 95% CI=-0.357 – -0.020, right: main effect of group, $F(2,44)=7.275$, $P=0.002$, partial $\eta^2=0.249$; early blind vs. sighted controls, Bonferroni $P=0.002$, 95% CI=-0.504 – -0.100), the bilateral supramarginal gyrus (left: main effect of group, $F(2,44)=7.747$, $P=0.001$, partial $\eta^2=0.260$; early blind vs. sighted controls, Bonferroni $P=0.006$, 95% CI=-0.685 – -0.097, late blind vs. sighted controls, Bonferroni $P=0.016$, 95% CI=-0.457 – -0.037, right: main effect of group, $F(2,44)=4.330$, $P=0.019$, partial $\eta^2=0.164$; early blind vs. sighted control, Bonferroni $P=0.033$, 95% CI=-0.565 – -0.018), and the bilateral lateral sensorimotor cortex (left: main effect of group, $F(2,44)=7.470$, $P=0.002$, partial $\eta^2=0.253$; early

blind vs. sighted controls, Bonferroni P=0.001, 95% CI=-0.589 – -0.127, early blind vs. late blind, Bonferroni P=0.012, 95% CI=-0.546 – -0.054, right: main effect of group, $F(2,44)=3.804$, P=0.030, partial η^2=0.147; early blind vs. sighted controls, Bonferroni P=0.026, 95% CI=-0.543 – -0.027). The early blind individuals also exhibited reduced rCVR compared to the late blind individuals in the left lateral sensorimotor cortex (early blind vs. late blind, Bonferroni P=0.013, 95% CI=-0.543 – -0.052). The late blind individuals showed reduced rCVR than sighted controls in the posterior cingulate cortex (main effect of group, $F(2,44)=3.722$, P=0.032, partial η^2=0.145; late blind vs. sighted controls, Bonferroni P=0.029, 95% CI=-0.529 – -0.022), the left intraparietal sulcus (late blind vs. sighted controls, Bonferroni P=0.010, 95% CI=-0.359 – -0.039), the left supramarginal gyrus (late blind vs. sighted controls, Bonferroni P=0.020, 95% CI=-0.455 – -0.031), the medial visual cortex (main effect of group, $F_{welch}(2,26.725)=4.348$, P=0.023, partial η^2=0.132; late blind vs. sighted controls, Games-Howell P=0.017, 95% CI=-0.5323 – -0.0449), and the occipital visual cortex (main effect of group, $F(2,44)=4.328$, P=0.019, partial η^2=0.164; late blind vs. sighted controls, Bonferroni P=0.016, 95% CI=-0.580 – -0.046). The distributions are represented using box plots and the outliers are plotted as plus signs. *corrected $P<0.05$, **corrected $P<0.01$, ***corrected $P<0.001$. Early blind: n=6, late blind: n=15, sighted controls: n=26.

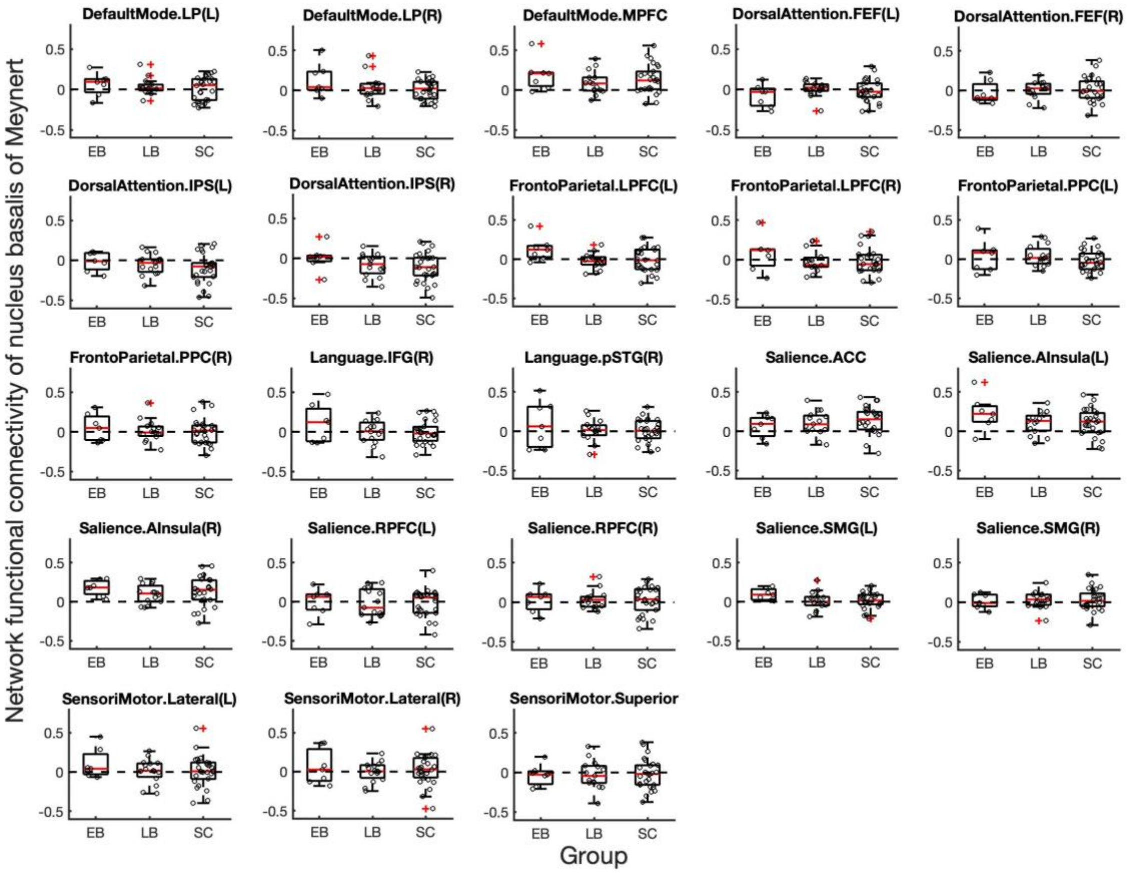

Supplementary Figure 4. Network functional connectivity between the nucleus basalis of Meynert and twenty-three cortical networks. We did not observe any group difference (all Ps>0.05) within any of the three default mode networks (bilateral lateral parietal cortex, medial prefrontal cortex), four dorsal attention networks (bilateral frontal eye fields, bilateral intraparietal sulcus), four frontoparietal networks (bilateral lateral prefrontal cortex, bilateral posterior parietal cortex), two language networks (right inferior frontal gyrus, right posterior superior temporal gyrus), seven salience networks (anterior cingulate cortex, bilateral anterior insular cortex, bilateral rostral prefrontal cortex, bilateral supramarginal gyrus), or three sensorimotor networks (bilateral lateral sensorimotor cortex, superior sensorimotor cortex). The distributions are represented using box plots and the outliers are plotted as plus signs. Early blind: n=7, late blind: n=15, sighted controls: n=26.

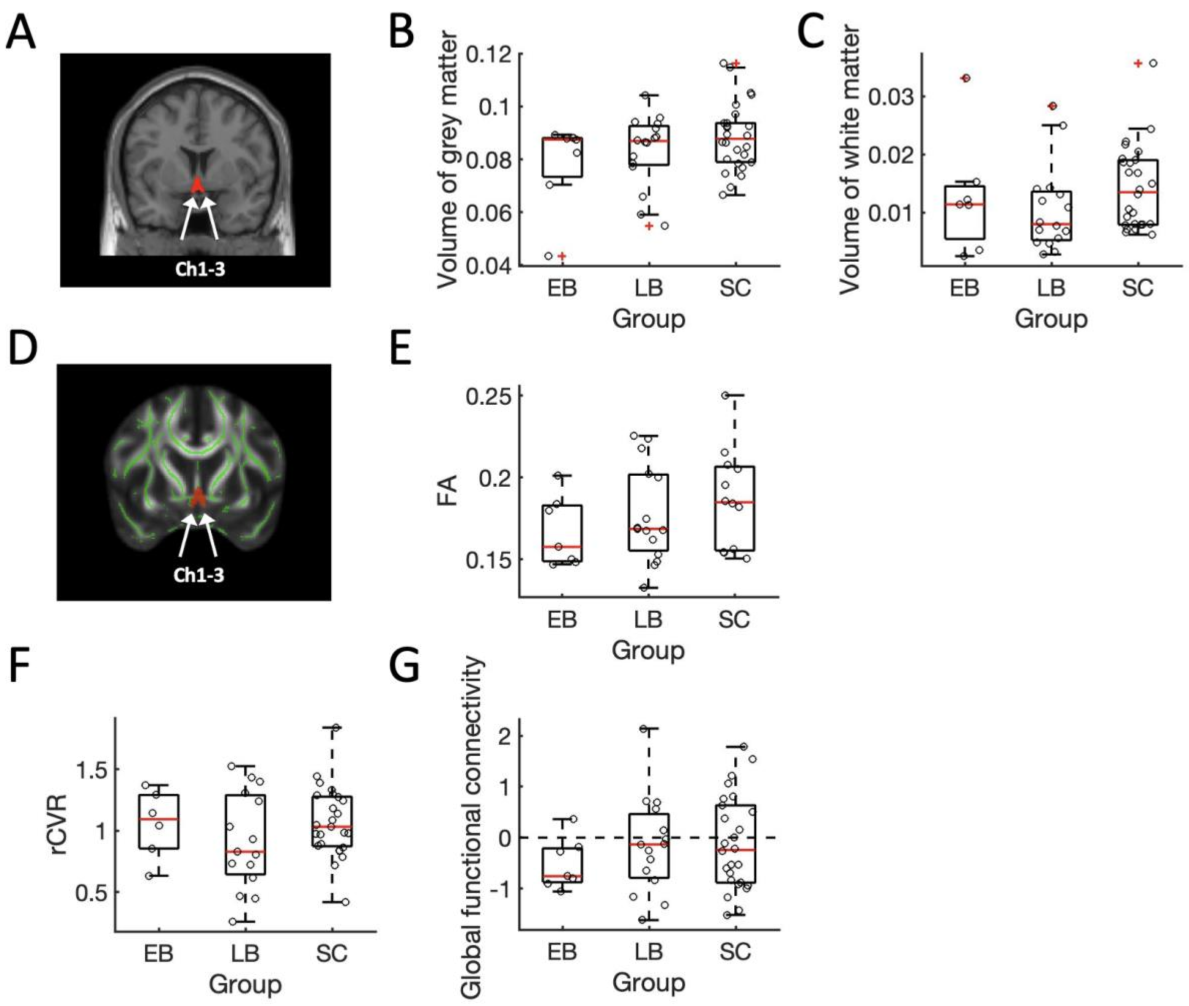

Supplementary Figure 5. Structural and physiological integrity in other basal forebrain regions (Ch1-3) beyond the nucleus basalis of Meynert. (A) Coronal view of the Ch1-3 (red) in T1-weighted MRI, which covers the magnocellular cell groups within the septum and the horizontal limb of the diagonal band. We observed no difference in the (B) grey matter volume (F(2,44)=0.873, *P*=0.425, partial η^2=0.038), or (C) white matter volume (F(2,44)=0.863, *P*=0.429, partial η^2=0.038) of the Ch1-3 across the early blind (EB), late blind (LB) individuals, and sighted controls (SC). (D) Coronal view of the Ch1-3 (red) overlaid on the mean FA skeleton (green). (E) Mean FA within the Ch1-3 is comparable across groups (F(2,29)=0.494, *P*=0.615, partial η^2=0.033). (F) The rCVR (F(2,43)=0.280, *P*=0.757, partial η^2=0.013), and (G) global functional connectivity (F(2,44)=0.636, *P*=0.534, partial η^2=0.028) of the Ch1-3 did not differ across groups. The distributions are represented using box plots and the outliers are plotted as plus signs. For the volumes of grey matter and white matter, early blind: *n*=7, late blind: *n*=16, sighted controls: *n*=26.

For FA, early blind: n=7, late blind: n=15, sighted controls: n=12. For rCVR, early blind: n=6, late blind: n=15, sighted controls: n=25. For global connectivity, early blind: n=7, late blind: n=15, sighted controls: n=26.

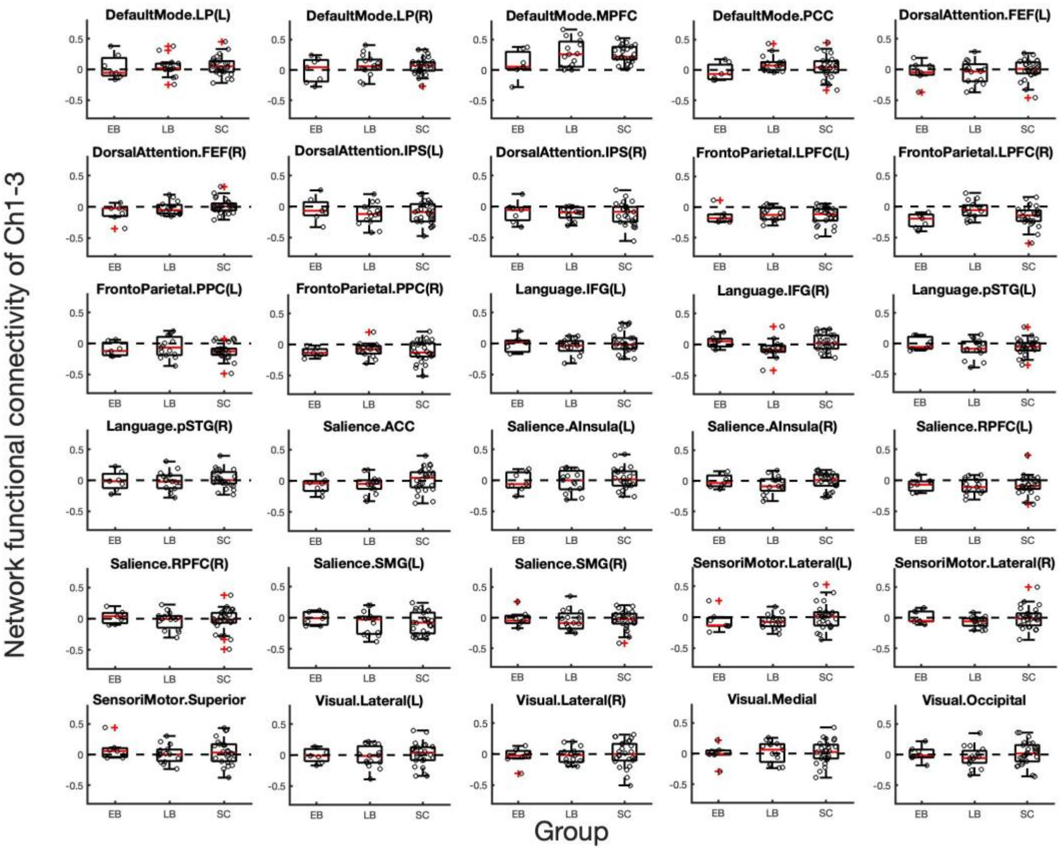

Supplementary Figure 6. Network functional connectivity between Ch1-3 and thirty cortical networks. We did not observe any difference between early blind (EB), late blind (LB) individuals, and sighted controls (SC) (all *P*s>0.05). The distributions are represented using box plots and the outliers are plotted as plus signs. Early blind: *n*=7, late blind: *n*=15, sighted controls: *n*=26.
